# Supplementary material for: Time-series transcriptome analysis identified differentially expressed genes in broiler chicken infected with mixed Eimeria species
Source: Front Genet. 2022 Aug 8;13:886781. doi: 10.3389/fgene.2022.886781 (PMC9393255; doi:10.3389/fgene.2022.886781)
Supplement: Supplementary file 2 [file DataSheet1.ZIP › 4dpi_KEGG.Gsea.1625100298584/KEGG_GLYCOSPHINGOLIPID_BIOSYNTHESIS_GLOBO_SERIES.html]

Details for gene set KEGG\_GLYCOSPHINGOLIPID\_BIOSYNTHESIS\_GLOBO\_SERIES[GSEA]

|  || Dataset | TMM\_4dpi\_gct\_format\_4dpi\_gct\_format.Class\_4dpi.cls #PC\_versus\_NC.Class\_4dpi.cls #PC\_versus\_NC\_repos |
| Phenotype | Class\_4dpi.cls#PC\_versus\_NC\_repos |
| Upregulated in class | 1 |
| GeneSet | KEGG\_GLYCOSPHINGOLIPID\_BIOSYNTHESIS\_GLOBO\_SERIES |
| Enrichment Score (ES) | 0.5478666 |
| Normalized Enrichment Score (NES) | 1.3505733 |
| Nominal p-value | 0.13086233 |
| FDR q-value | 0.51530737 |
| FWER p-Value | 1.0 |
Table: GSEA Results Summary

  

Fig 1: Enrichment plot: KEGG\_GLYCOSPHINGOLIPID\_BIOSYNTHESIS\_GLOBO\_SERIES      
 Profile of the Running ES Score & Positions of GeneSet Members on the Rank Ordered List

  

| SYMBOL | TITLE | RANK IN GENE LIST | RANK METRIC SCORE | RUNNING ES | CORE ENRICHMENT || 1 | FUT9 | na | 20 | 2.261 | 0.3250 | Yes |
| 2 | B3GALT5 | na | 110 | 1.594 | 0.5479 | Yes |
| 3 | ST3GAL1 | na | 4333 | 0.139 | 0.2161 | No |
| 4 | ST8SIA1 | na | 6635 | -0.057 | 0.0326 | No |
| 5 | GBGT1 | na | 8066 | -0.186 | -0.0597 | No |
| 6 | HEXB | na | 8235 | -0.202 | -0.0446 | No |
| 7 | B3GALNT1 | na | 8350 | -0.213 | -0.0232 | No |
| 8 | ST3GAL2 | na | 8365 | -0.215 | 0.0067 | No |
| 9 | HEXA | na | 8634 | -0.243 | 0.0194 | No |
| 10 | A4GALT | na | 9999 | -0.415 | -0.0343 | No |
| 11 | NAGA | na | 10744 | -0.548 | -0.0172 | No |
| 12 | GLA | na | 11566 | -0.849 | 0.0370 | No |
Table: GSEA details [plain text format]

  

Fig 2: KEGG\_GLYCOSPHINGOLIPID\_BIOSYNTHESIS\_GLOBO\_SERIES      
 Blue-Pink O' Gram in the Space of the Analyzed GeneSet

  

Fig 3: KEGG\_GLYCOSPHINGOLIPID\_BIOSYNTHESIS\_GLOBO\_SERIES: Random ES distribution      
 Gene set null distribution of ES for **KEGG\_GLYCOSPHINGOLIPID\_BIOSYNTHESIS\_GLOBO\_SERIES**
